# Evaluation of Pulmonary Reperfusion Injury in Rats Undergoing Mesenteric Ischemia and Reperfusion and Protective Effect of Postconditioning on this Process

**DOI:** 10.5935/1678-9741.20150067

**Published:** 2015

**Authors:** Carlos Henrique Marques dos Santos, Ricardo Dutra Aydos, Ed Nogueira Neto, Luciana Nakao Odashiro Miiji, Pedro Carvalho Cassino, Isadora Ishaq Alves, Nádia Meneguesso Calheiros, Milena Garcia

**Affiliations:** 1 Department of Surgery. Universidade Federal do Mato Grosso do Sul (UFMS), Campo Grande, MS, Brazil.; 2 Department of Pathology. Universidade Federal do Mato Grosso do Sul (UFMS), Campo Grande, MS, Brazil.; 3 Universidade Federal do Mato Grosso do Sul (UFMS), Campo Grande, MS, Brazil.; 4 Faculdade de Medicina da Universidade Federal do Mato Grosso do Sul (FAMED-UFMS), Campo Grande, MS, Brazil.

**Keywords:** Ischemic Postconditioning, Ischemia, Lung Injury, Reperfusion Injury, Intestinal Mucosa

## Abstract

**INTRODUCTION:**

Some publications have demonstrated the presence of lung reperfusion injury in mesenteric ischemia and reperfusion (I/R), but under to diverse methods. Postconditioning has been recognized as effective in preventing reperfusion injury in various organs and tissues. However, its effectiveness has not been evaluated in the prevention of lung reperfusion injury after mesenteric ischemia and reperfusion.

**OBJECTIVE:**

To evaluate the presence of pulmonary reperfusion injury and the protective effect of ischemic postconditioning on lung parenchyma in rats submitted to mesenteric ischemia and reperfusion.

**METHODS:**

Thirty Wistar rats were distributed into three groups: group A (10 rats), which was held mesenteric ischemia (30 minutes) and reperfusion (60 minutes); group B (10 rats), ischemia and reperfusion, interspersed by postconditioning with two alternating cycles of reperfusion and reocclusion, for two minutes each; and group C (10 rats), ischemia and reperfusion interleaved by postconditioning with four alternating cycles of reperfusion and reocclusion of 30 seconds each. Finally, it was resected the upper lung lobe for histological analysis.

**RESULTS:**

There were mild lung lesions (grade 1) in all samples. There was no statistical difference between groups 1 and 2 (*P*>0.05).

**CONCLUSION:**

The mesenteric ischemia and reperfusion in rats for thirty and sixty minutes, respectively, caused mild reperfusion injury in lung. Postconditioning was not able to minimize the remote reperfusion injury and there was no difference comparing two cycles of two minutes with four cycles of 30 seconds.

**Table:** 

**Abbreviations, acronyms & symbols**	
I/R	= Ischemia and reperfusion
IPC	= Ischemic postconditioning
ROS	= Reactive oxygen species
TNF	= Tumor necrosis factor

## INTRODUCTION

Since 1986, when Parks & Granger^[1]^ demonstrated the harmful effects of toxic reactive oxygen species (ROS) produced during reperfusion, many researches have been developed in search of an experimental model that could minimize this process in order to reduce the cellular and organic damage ischemia and reperfusion (I/R)^[2,3]^.

The best results ever published in controlling the production of ROS were obtained with the ischemic preconditioning, as numerous publications that followed Murry et al.^[4]^, including the mesenteric I/R. However, there is little applicability in clinical situations for the ischemic preconditioning, for example, in the acute abdomen with mesenteric ischemia, when the diagnosis is made when the ischemia already exists and it's impossible to use this method.

In 2003, Zhao et al.^[2]^ presented the concept of ischemic postconditioning (IPC), which consists of making one or more short cycles of reperfusion followed by one or more short cycles of ischemia, immediately after ischemia period and before to give permanent reperfusion.

In experimental model, there is already evidence of the IPC protective effect on the intestinal mucosa of rats undergoing mesenteric I/R^[4]^, and recently, IPC was able to minimize the severity of liver injury in rats undergoing I/R^[5]^. Several published experiments examined the effects of IPC in other organs and tissues, among which may be mentioned Darling et al.^[6]^ in which the IPC was able to minimize the infarction area of myocardium in rabbits. Tang et al.^[7]^ demonstrated the effectiveness of IPC in preventing injuries resulting from the coronary I/R in rats, since the ischemia time did not exceed 45 minutes. Huang et al.^[3]^ demonstrated that IPC were preventing tissue damage in the spinal cord of rats subjected to I/R. Santos et al.^[8]^ showed that the ischemic preconditioning and IPC were able to minimize the tissue injury in the intestines of rats subjected to mesenteric I/R process.

However, reperfusion injury can not only affect the ischemic and then reperfused organ, but can also damage remote organs, such as pulmonary edema presented after some I/R process. The restoration of hemodynamic stability after a circulatory shock is a clinical situation of I/R, with the possibility of extensive damage because the amount of tissues involved^[9]^.

It was believed that the lung was more resistant to ischemic injury than other organs. Two factors contribute to either: the presence of bronchial circulation beyond the pulmonary circulation and the fact that the interruption of pulmonary blood flow is not accompanied by hypoxia, since the alveolar ventilation is maintained. The lung can be considered as the only organ that can undergo ischemia without hypoxia^[10]^.

However, in recent years some evidence has emerged that the lung can not be completely immune to reperfusion injury maintained despite the gas exchange, since the ROS act systemically. In surgeries with temporary occlusion of the aorta, pulmonary edema constitutes a common complication, by a multifactorial pathway, including reperfusion injury. Already during ischemia, there is an increase in pulmonary arterial pressure, a factor that may favor the formation of edema in the lungs. This increased resistance in the pulmonary circulation is a result in part of a larger blood flow, due to its redistribution to the territory above the occlusion, and increased left ventricular end-diastolic volume, which emptying is impaired by increasing the aortic occlusion imposed on the afterload^[11]^.

Mesenteric I/R is associated with the production of other inflammatory mediator, the tumor necrosis factor (TNF). The intestinal mucosal injury by I/R allows the release of endotoxin to the portal circulation, inducing TNF production by hepatic macrophages. The increased of TNF in the systemic circulation can lead to inflammatory lung injury characterized by neutrophil accumulation. This sequence of events was demonstrated by Caty et al.^[12]^ in a model of I/R by temporary occlusion of the superior mesenteric artery in rats. After reperfusion, there was increase in endotoxin levels in portal venous blood and TNF in the systemic circulation. At the same time there was accumulation of neutrophils in the lungs and increased pulmonary capillary permeability.

The question, however, is how long the periods of ischemia and reperfusion must last to cause reperfusion injury not only in the intestine but also in the lung. Furthermore, the mechanisms used for the prevention of reperfusion injuries, such as IPC, would be capable of not only preventing intestinal tissue damage but also the remote lesions.

Thus, considering that there are different experimental models of I/R, is too relevant and is the aim of this study to verify if a model that causes intestinal damage can also cause lung reperfusion injury, and if the IPC can minimize such lesions.

### Objective

To assess the presence of pulmonary reperfusion injury and the protective effect of ischemic postconditioning on lung parenchyma in rats undergoing mesenteric ischemia and reperfusion.

## METHODS

The study was approved by the Ethics Committee for Animal Experimentation of the Universidade Federal do Mato Grosso do Sul and was based on ethical principles advocated by the Brazilian College of Animal Experimentation.

**Fig. 1:**
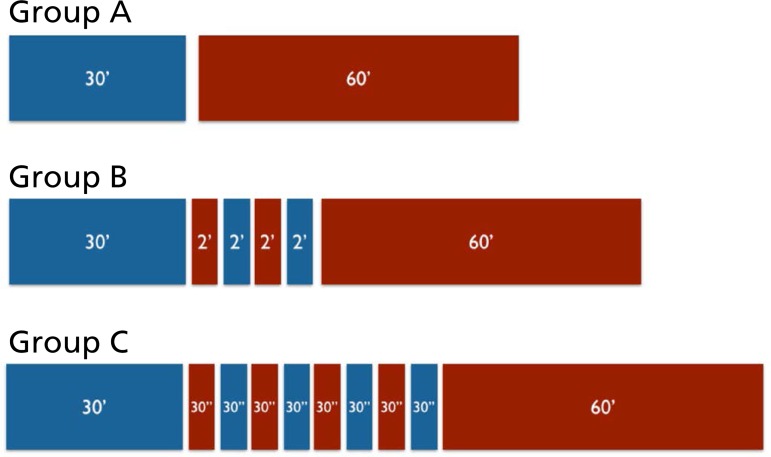
Schematic demonstration of periods of ischemia and reperfusion in groups A, B and C (the numbers correspond to the time in minutes; blue: ischemia; red: reperfusion).

It was used 30 rats (*Rattus norvegicus*) of the Wistar albino strain, adults, males, weighing 270-350 grams, with an average of 305 grams, from the vivarium of the Federal University of Mato Grosso do Sul. The animals were distributed in following groups (Figure 1):

- Group A - Ischemia and Reperfusion (IR):

Ten rats underwent intestinal ischemia for 30 minutes by occlusion of the cranial mesenteric artery with a vascular clamp, followed by reperfusion for 60 minutes for removal of the clamp.

- Group B - ischemic postconditioning 1 (IPC-1):

Ten rats underwent ischemia for 30 minutes by occlusion of the cranial mesenteric artery with vascular clamp and reperfusion for 60 minutes. Among ischemia and reperfusion were performed two reperfusion cycles (two minutes each) interleaved by two ischemia cycles (two minutes each).

- Group C - ischemic postconditioning 2 (IPC-2):

Ten rats underwent ischemia for 30 minutes by occlusion of the cranial mesenteric artery with vascular clamp and reperfusion for 60 minutes. Among ischemia and reperfusion were performed four cycles of reperfusion (30 seconds each) interleaved by four ischemia cycles (30 seconds each).

The animals were weighed on an electronic precision scale and anesthetized by intraperitoneal injection of solution 2:1 of Ketamine hydrochloride (Cetamin^®^), 50 mg/ml, and Xylazine hydrochloride (Xilazin^®^), 20 mg/ml, respectively, at a dose of 0.1 ml/100g. The rats were considered anesthetized after being found loss of eyelid reflex, loss of response to mechanical stimuli, loss of righting reflex and withdrawing member after painful stimulus caused by hold.

After, the anesthetized rats underwent abdominal trichotomy and were positioned to the operating table in the supine position with the four members in abduction. Then a longitudinal median laparotomy of about four centimeters was performed, exteriorization of the small intestine, identification and dissection of the cranial mesenteric artery.

In group A, the cranial mesenteric artery was occluded with atraumatic vascular clamp which remained for 30 minutes (ischemic phase). After placing the clamp, the small intestine was repositioned in the abdominal cavity and the wound was closed with a continuous suture of the skin with nylon monofilament (mononylon^®^) 4-0. After the stage of ischemia, the abdominal wall was opened again by removing the suture and the vascular clamp was removed, beginning the reperfusion phase, lasting 60 minutes. Started the reperfusion, the abdomen was again closed by continuous suture of the skin with nylon monofilament 4-0 until the end of the experiment.

In group B, after ischemia phase (30 minutes), IPC was performed through two cycles of ischemia, lasting two minutes each (removal of the clamp of the cranial mesenteric artery), interspersed with two cycles of ischemia also lasting two minutes each (application of atraumatic vascular clamp in the cranial mesenteric artery). After, there was the reperfusion for 60 minutes.

In group C was performed ischemia phase (30 minutes) and reperfusion (60 minutes). Preceding the reperfusion was performed IPC through four cycles of reperfusion (removal of atraumatic vascular clamp of the cranial mesenteric artery) lasting 30 seconds each, interspersed with four cycles of ischemia (occlusion of the cranial mesenteric artery by atraumatic vascular clamp), also lasting 30 seconds each.

After completion of reperfusion in all groups, thoracotomy was performed and resection of the right upper lung lobe, which is washed with saline and then placed in a 10% solution of formaldehyde for subsequent histological analysis.

The animals were euthanized by increasing the anesthesia.

The lung segments resected, after fixation in 10% formaldehyde solution, they were subjected to histological processing for 12 hours in automatic histotechnical (AUTOTECHNICON^TM^ DUO-TECHNICON CORPORATION - MOD 2A). After processing were embedded in paraffin and subjected to histological sections in macrometry (Leica^TM^ 2025) each 4 µm. The slides were stained with hematoxylin-eosin and analyzed by optical microscopy (microscope Nikon^TM^ E200) by the pathologist without prior knowledge of this on the group belonging to each rat.

The laminas made from the resected lung segments were analyzed according to Sizlan et al.^[13]^ classification:

- Grade 0: no change.
- Grade 1: mild neutrophilic infiltrate and mild to moderate interstitial congestion.
- Grade 2: moderate neutrophilic infiltrate, perivascular edema formation and partial destruction of the lung architecture.
- Grade 3: dense neutrophilic infiltrate and complete destruction of lung parenchyma.

The results were analyzed statistically, applying the nonparametric Kruskal-Wallis test, and established a significance level of *P*<0.05. It was used the 5.4 Bioestat program.

## RESULTS

After the histological analysis of the degree of lung injury, were found the following results (Table 1).

**Table 1:** Results of histological analysis of the reperfusion injury of lung according to Sizlan et al.^[13]^.

Rats	Degree of lesion		
	Group IR	Group IPC 1	Group IPC 2
1	1	1	1
2	1	1	1
3	1	1	1
4	1	1	1
5	1	1	1
6	1	1	1
7	1	1	1
8	1	1	1
9	1	1	1
10	1	1	1
Average	1	1	1

Group IR=ischemia and Reperfusion; Group IPC1=ischemic Postconditioning 1 (two cycles of reperfusion and ischemia lasting two minutes each); Group IPC 2=ischemic Postconditioning 2 (four cycles of reperfusion and ischemia lasting 30 seconds each) Note: P>0.05 between groups A, B and C.

## DISCUSSION

The intestinal I/R process can cause severe tissue damage and increased intestinal permeability, depending on the time and intensity of this process. He et al.^[14]^ demonstrated that morphological changes occur as injury mucosa, villous erosion, necrosis, interstitial congestion in the lamina propria, edema, inflammation and submucosal hemorrhage. This increased intestinal permeability leading to bacterial translocation which can contribute to a systemic inflammatory response mediated by cytokines. The HMGB1 protein is an endogenous ligand that plays an important role in this process and is directly related to sepsis and increased mortality. In the early stages of I/R, there is an immediate increase of HMGB1 that continues increasing slowly during reperfusion and can lead to accumulation of neutrophils and pulmonary edema^[14]^.

In addition to the cytokines, the activation of the immune system by ischemic bowel produces TNF-α and IL-6. Systemic inflammatory response after I/R activates neutrophils, which are sequestered in the pulmonary microcirculation with the consequent increase in endothelial and epithelial permeability, extravasation of fluids and proteins, leukocytes sequestration and increased injury to the endothelium of the pulmonary capillaries^[15]^. Certainly this process will be more intense and damaging as the I/R has a longer duration, resulting in increased intestinal permeability and greater local and remote inflammatory process. This could justify the fact that in this study we observed mild lung injury, while other publications using longer periods of I/R has demonstrated increased lung damage.

This fact was noted in the publication of Guido et al.^[16]^ in which the authors performed ischemia for 45 minutes and reperfusion for two and 24 hours, finding lung injury histologically moderate to intense. The authors observed that the reperfusion period is a crucial factor for the appearance of the lesion at a distance, especially the lung, as evidenced by the lower accumulation of neutrophils in the lung parenchyma after two hours of reperfusion compared to 24 hours when there was higher concentration of these cells.

Using 30 minutes of ischemia and three hours of reperfusion, Lapchak et al.^[17]^ showed no statistical difference in the concentration of neutrophils in the lungs of rats undergoing mesenteric I/R compared to the SHAM group, which may be related to a lower period of ischemia than in other publications that noted higher neutrophil concentration.

Several studies have confirmed the presence of lung injury after I/R, such as the publication of Wang et al.^[18]^ in which the authors applied mesenteric I/R for 60 and 120 minutes, respectively, observing moderate lung injury. Also Sotoudeh et al.^[19]^ obtained moderate degree of lung injury performing a study with ischemia for two hours of rats' femoral artery and 24 hours of reperfusion. Although there was a higher degree of lung injury in this study, one must consider the great difference of the adopted periods of I/R as well as the organ subjected to this process.

He et al.^[20]^ achieved average 2.5 of lung injury using the classification proposed by Sizlan et al.^[13]^ in intestinal I/R. These authors used Sprague-Dawley rats with periods of I/R higher than those employed here, 45 and 120 minutes, respectively.

Idrovo et al.^[15]^ also performed mesenteric I/R in Sprague Dawley rats, however, with ischemia lasting 90 minutes and reperfusion four hours. They evaluated the pulmonary repercussion of this process and observed a moderate to severe injury by histological classification. It should here point out that the difference with the results obtained here is probably due to long periods of I/R used by these authors.

Thomaz Neto et al.^[21]^ had already shown that the mesenteric I/R process causes mild lung injury, and that this can be minimized by ischemic preconditioning. However, this method of prevention has limited clinical applicability, since the identifying mesenteric ischemia no further opportunity for their use. In this study were used periods of I/R of 30 minutes. The IPC would have greater clinical applicability in situations like this, since this has been shown to be effective in preventing or decreasing reperfusion injury in various organs investigated, including the intestine. However, despite its proven efficacy, there are no studies in the scientific literature reviewed of IPC on prevention of remote injury in the mesenteric I/R process. In this study there was no difference between the group submitted only to the I/R and the two groups treated by IPC.

Recently, Dorsa et al.^[22]^ had demonstrated a protective effect of IPC on lung of rats undergoing aortic ischemia and reperfusion process. They have used also 30 minutes of ischemia and 60 minutes of reperfusion, but it was made by aortic occlusion, differently of our research. Another difference relates to number of cycles, once they used three cycles of IPC and we applied just two cycles of two minutes each.

The IPC protective mechanism in the mesenteric I/R process is still not entirely clear, but there is evidence that IPC may be related to a significant decrease in the levels of malondialdehyde and products related to lipid peroxidation. These observations suggest a reduction in the production of ROS and less injury mediated by oxidants with IPC^[8]^.

The peak of ROS production occurs between the first and seventh minute after initiation of reperfusion, although such substances are detectable in later periods. An abundant production of ROS during this initial phase of reperfusion has been implicated as a major factor in the pathogenesis of tissue injury^[23]^. The IPC acts at this stage, through a pathway not fully understood, probably reducing the production of ROS by the gradual release of oxygen to tissues^[24]^.

Thus, considering what is known today about the mechanism of action of the IPC, this also supposed to decrease injuries to the distance as in the case of the lung. However, we must consider that there is still much to discover regarding the pathophysiology of the process and about the many mechanisms and drugs used to prevent reperfusion injury. In analyzing the articles cited here, although we have observed a considerable number of publications showing ways to prevent remote lung injury, there is no standardization in methods, making it very difficult to compare them. Besides the difference between animals used, the variation in the periods of mesenteric ischemia and reperfusion is very large, and therefore, this should lead to a variety of lung injury in intensity.

The data in our publication leave us the information that 30 and 60 minutes of mesenteric I/R cause mild lung injury, unlike what happens in the gut, where literature shows that there is high grade of injury^[8]^. That means that the lung can withstand more than the intestine subjected to I/R. Although IPC has not been helpful in minimizing lung reperfusion injury in this study, there is a doubt as to its effectiveness in the case of most lung damage, and this should be studied in future research.

## CONCLUSION

The mesenteric ischemia and reperfusion in rats for thirty and sixty minutes, respectively, caused mild reperfusion lung injury. Ischemic postconditioning was not able to minimize the pulmonary reperfusion injury and there was no difference between two cycles of two minutes with four cycles of 30 seconds.

**Table:** 

**Authors' roles & responsibilities**	
CHMS	Analysis/interpretation of data; study design; implementation of projects/experiments; manuscript writing/critical review of its content; final approval of the manuscript
RDA	Final approval of the manuscript; study design
ENN	Conduct of operations/experiments; final approval of the manuscript
LNOM	Analysis/interpretation of data; final approval of the manuscript
PCC	Conduct of operations/experiments; final approval of the manuscript
IIA	Statistical analysis; implementation of projects/ experiments; final approval of the manuscript
NMC	Analysis/interpretation of data; implementation of projects/experiments; final approval of the manuscript
MG	Analysis/interpretation of the data; final approval of the manuscript

## References

[1] Parks DA, Granger DN (1986). Contributions of ischemia and reperfusion to mucosal lesion
formation. Am J Physiol.

[2] Zhao ZQ, Corvera JS, Halkos ME, Kerendi F, Wang NP, Guyton RA (2003). Inhibition of myocardial injury by ischemic postconditioning
during reperfusion: comparison with ischemic preconditioning. Am J Physiol Heart Circ Physiol.

[3] Huang H, Zhang L, Wang Y, Yao J, Weng H, Wu H (2007). Effect of ischemic post-conditioning on spinal cord
ischemic-reperfusion injury in rabbits. Can J Anaesth.

[4] Murry CE, Jennings RB, Reimer KA (1986). Preconditioning with ischemia: a delay of lethal cell injury in
ischemic myocardium. Circulation.

[5] Santos CH, Pontes JC, Miiji LN, Nakamura DI, Galhardo CA, Aguena SM (2010). Postconditioning effect in the hepatic ischemia and reperfusion
in rats. Acta Cir Bras.

[6] Darling CE, Jiang R, Maynard M, Whittaker P, Vinten-Johansen J, Przyklenk K (2005). Postconditioning via stuttering reperfusion limits myocardial
infarct size in rabbit hearts: role of ERK1/2. Am J Physiol Heart Circ Physiol.

[7] Tang XL, Sato H, Tiwari S, Dawn B, Bi Q, Li Q (2006). Cardioprotection by postconditioning in conscious rats is limited
to coronary occlusions < 45 min. Am J Physiol Heart Circ Physiol.

[8] Santos CHM, Pontes JCDV, Gomes OM, Miiji LNO, Bispo MAF (2009). Evaluation of ischemic postconditioning effect on mesenteric
ischemia treatment. Experimental study in rats. Rev Bras Cir Cardiovasc.

[9] Pinheiro BV, Holanda MA, Araújo FG, Romaldini H (1999). Lesão pulmonar de reperfusão. J Pneumol.

[10] Heffner JE, Fracica P, Steudel W, Zapol WM (1996). Ischemia-reperfusion edema of the lung: advances in mechanistic
understanding. Nitric oxide and radicals in the pulmonary vasculature.

[11] Gelman S (1995). The pathophysiology of aortic cross-clamping and
unclamping. Anesthesiology.

[12] Caty MG, Guice KS, Oldham KT, Remick DG, Kunkel SI (1990). Evidence for tumor necrosis factor-induced pulmonary
microvascular injury after intestinal ischemia-reperfusion
injury. Ann Surg.

[13] Sizlan A, Guven A, Uysal B, Yanarates O, Atim A, Oztas E (2009). Proanthocyanidin protects intestine and remote organs against
mesenteric ischemia/reperfusion injury. World J Surg.

[14] He GZ, Zhou KG, Zhang R, Wang YK, Chen XF (2012). Impact of intestinal ischemia/reperfusion and lymph drainage on
distant organs in rats. World J Gastroenterol.

[15] Idrovo JP, Yang WL, Jacob A, Aziz M, Nicastro J, Coppa GF (2014). AICAR attenuates organ injury and inflammatory response after
intestinal ischemia and reperfusion. Mol Med.

[16] Guido BC, Zanatelli M, Tavares-de-Lima W, Oliani SM, Damazo AS (2013). Annexin-A1 peptide down-regulates the leukocyte recruitment and
up-regulates interleukin-10 release into lung after intestinal
ischemia-reperfusion in mice. J Inflamm (Lond).

[17] Lapchak PH, Ioannou A, Rani P, Lieberman LA, Yoshiya K, Kannan L (2012). The role of platelet factor 4 in local and remote tissue damage
in a mouse model of mesenteric ischemia/reperfusion injury. PLoS One.

[18] Wang J, Qiao L, Li S, Yang G (2013). Protective effect of ginsenoside Rb1 against lung injury induced
by intestinal ischemia-reperfusion in rats. Molecules.

[19] Sotoudeh A, Takhtfooladi MA, Jahanshahi A, Asl AH, Takhtfooladi HA, Khansari M (2012). Effect of N-acetylcysteine on lung injury induced by skeletal
muscle ischemia-reperfusion. Histopathological study in rat
model. Acta Cir Bras.

[20] He XH, Li QW, Wang YL, Zhang ZZ, Ke JJ, Yan XT (2015). Transduced PEP-1-heme oxygenase-1 fusion protein reduces remote
organ injury induced by intestinal ischemia/reperfusion. Med Sci Monit.

[21] Thomaz FJ, Koike MK, Abrahão MS, Carillo F, Pereira RK, Machado JL (2013). Ischemic preconditioning attenuates remote pulmonary inflammatory
infiltration of diabetic rats with an intestinal and hepatic
ischemia-reperfusion injury. Acta Cir Bras.

[22] Dorsa RC, Pontes JCDV, Antoniolli ACB, Silva GVR, Benfatti RA, Santos CHM (2015). Effect of remote ischemic postconditioning in inflammatory
changes of the lung parenchyma of rats submitted to ischemia and
reperfusion. Rev Bras Cir Cardiovasc.

[23] Sun HY, Wang NP, Kerendi F, Halkos M, Kin H, Guyton RA (2005). Hypoxic postconditioning reduces cardiomyocyte loss by inhibiting
ROS generation and intracellular Ca2+ overload. Am J Physiol Heart Circ Physiol.

[24] Lim SY, Davidson SM, Hausenloy DJ, Yellon DM (2007). Preconditioning and postconditioning: the essential role of the
mitochondrial permeability transition pore. Cardiovasc Res.

